# Development of Publicly Available Forensic DNA Sequence Mixture Data

**DOI:** 10.3390/genes16030333

**Published:** 2025-03-12

**Authors:** Erica L. Romsos, Kevin M. Kiesler, Carolyn R. Steffen, Lisa A. Borsuk, Sarah Riman, Lauren E. Mullen, Jodi A. Irwin, Peter M. Vallone, Katherine B. Gettings

**Affiliations:** 1 National Institute of Standards and Technology, 100 Bureau Drive, Gaithersburg, MD 20899, USA; erica.romsos@nist.gov (E.L.R.); kevin.kiesler@nist.gov (K.M.K.); carolyn.steffen@nist.gov (C.R.S.); lisa.borsuk@nist.gov (L.A.B.); sarah.riman@nist.gov (S.R.); lauren.mullen@nist.gov (L.E.M.); peter.vallone@nist.gov (P.M.V.); 2 Federal Bureau of Investigation Laboratory, 2501 Investigation Parkway, Quantico, VA 22135, USA; jairwin@fbi.gov

**Keywords:** forensic DNA, sequencing, training data, validation, bioinformatics, mixtures

## Abstract

*Background*: In 2018, the Next-Generation Sequencing Committee of SWGDAM queried bioinformatic and statistical interpretation method developers regarding data needs for the development of sequence-based probabilistic genotyping software. *Methods*: Based on this engagement, a set of 74 mixture samples was conceived and created using 11 single-source samples. The allelic overlap among these samples was evaluated and sample combinations of varying complexity were selected, aiming to represent the variability observed in forensic casework. *Results*: The samples were distributed into a 96-well plate design containing several features: (1) three-person mixtures of 1% to 5% minor components in triplicate with varying levels of input DNA to provide information on sensitivity and reproducibility, (2) three-person mixtures containing degraded DNA of either only the major contributor or all three contributors, (3) four- and five-person mixtures with varying ratios and donors, (4) a single-source dilution series. *Conclusions*: Mixture samples were prepared and have been sequenced thus far with three commercially available kits targeting forensic short tandem repeat (STR) and single nucleotide polymorphism (SNP) markers, with FASTQ data files and metadata publicly available at doi.org/10.18434/M32157.

## 1. Introduction

Over the past 10 years, commercial next-generation sequencing assays have been introduced to the forensic community which target the short tandem repeat (STR) markers commonly found in the traditional Capillary Electrophoresis (CE, PCR fragment length-based) STR typing methods and varying combinations of additional single nucleotide polymorphism (SNP) markers [1,2,3,4]. Some STR loci exhibit a marked increase in observed alleles and heterozygosity via sequencing of population samples, and this additional information could be valuable in mixture interpretation. Also, over the past 10 years, the forensic community has been moving away from manual mixture interpretation methods and toward probabilistic genotyping approaches for traditional CE STR typing results [5,6]. While STR sequences can be converted back to length-based alleles, facilitating the use of existing probabilistic genotyping mixture interpretation methods, this approach is not ideal for several reasons. First, reducing the sequences to length-based alleles eliminates the information gained from sequencing and results in a less informed mixture interpretation (i.e., an impact on the assigned LR value for a contributor and information that can inform the estimation of the number of contributors in a mixture). Second, the length-based assay performance parameters implemented in the software may not be appropriate for a sequencing assay due to the multiple steps of library preparation and normalization [7,8,9,10]. While publicly available datasets such as the PROVEDIt database containing mixture data exist, this database is limited to CE STR typing results and does not house NGS datasets [11]. Existing large-scale repositories of publicly available human DNA sequence data consist of whole genome sequencing (WGS) or genotyping array data [12,13,14]. While some researchers have developed tools and benchmark reference genomes for exploring STRs in WGS data [15,16,17], these data would not be suitable for developing interpretation methods for the targeted assays used in forensic DNA testing; furthermore, these sequence data repositories contain data from individuals rather than mixtures of individuals. As STR sequence mixture interpretation methods continue to evolve, improving current models with a deeper understanding of assay performance and the impact of workflow decisions, a call for additional data to support such efforts exists [18,19,20,21].

Responding to this need, the Next-Generation Sequencing Committee of the Scientific Working Group on DNA Analysis Methods (SWGDAM) queried bioinformatic and statistical interpretation method developers to determine what types of data would be useful in advancing this method/technology. Based on this feedback, a set of mixture samples was conceived to be composed of three-, four-, and five-person mixtures and run with commercially available STR sequencing assays, the data from which would be publicly available. The mixture ratios selected for this work were a subset of a larger CE-based probabilistic genotyping validation study performed at the FBI laboratory, chosen pragmatically as an initial step in addressing the broad factor space of concentrations, allelic combinations, and mixture ratios.

The eleven single-source samples from the NIST Forensic DNA Open Dataset were examined for allelic overlap by length at 23 autosomal STR (auSTR) loci, and sample combinations of varying complexity were chosen. Three combinations of three-person mixtures were designed, along with six combinations each of four- and five-person mixtures. The mixtures were prepared using single-source samples that were quantified using digital PCR (dPCR) methods. The final stock solution of each mixture was also confirmed using dPCR methods.

To confirm the expected ratio of the contributors, a quality check of the prepared mixtures was performed by genotyping with PowerPlex Fusion 6C, analyzing the resulting CE-based STR profiles with STRmix v2.8.0, and comparing the resulting mixture ratios with the expected ratios. The samples were then sequenced with three commercially available sequencing kits targeting various combinations of STR and SNP markers: ForenSeq DNA Signature Prep Kit with DNA Primer Mix B (DPMB), Precision ID GlobalFiler NGS Panel v2, and PowerSeq 46GY Kit.

The FASTQ data files for these mixtures and single-source samples for all three sequencing kits, and corresponding metadata, along with .hid files from the STR genotyping are publicly available at doi.org/10.18434/M32157.

## 2. Materials and Methods

### 2.1. DNA Samples

Eleven donor buffy coat samples were purchased from Interstate Blood Bank Inc. (Memphis, TN, USA) under the approval of the NIST Research Protections Office. These samples support the NIST Forensic DNA Open Dataset (doi.org/10.18434/M32157), which contains previously generated data for forensic DNA targets (including single-source sample data from ForenSeq DNA Signature Prep Kit with DNA Primer Mix B (DPMB) (QIAGEN, Hilden, Germany), Precision ID GlobalFiler NGS Panel v2 (Thermo Fisher Scientific, Waltham, MA, USA), and PowerSeq 46GY Kit (Promega, Madison, WI, USA)). The extracted samples were quantified and underwent preliminary screening with STR genotyping for concordance testing to assess for abnormalities (e.g., high stutter, null alleles, tri alleles, peak height imbalance, etc.) and allelic overlap, as well as to determine an optimal degradation protocol (results not included). Samples were extracted as described in Romsos et al. [11] and concentrations were determined through digital PCR (dPCR) using the NEIF assay, which was determined to be a single-copy target of 67 base pairs in length using the conditions outlined in Romsos et al. [22,23,24]. Working stocks at 10 ng/µL and 1 ng/µL were prepared based on the dPCR values.

### 2.2. DNA Degradation

DNA degradation was performed by sonicating 130 µL of the sample with the Covaris S2 sonicator (Covaris, Wolburn, MA, USA) with the following settings: duty cycle = 10%, intensity = 10, cycles/burst = 100, at a temperature of ≈6 °C, with a varying number of 60 s cycles to achieve a 4 min, 6 min, 8 min, 15 min, or 30 min treatment. The degree of DNA degradation was evaluated with the D1000 DNA High Sensitivity ScreenTape on the 4150 TapeStation (Agilent, Santa Clara, CA, USA) following the manufacturer’s recommended protocol to determine the optimal time for sonication. The final samples were sonicated in a 130 µL volume for 15 min, which was determined to be optimal due to the fragmentation size being in the range relevant to STR analysis (e.g., 100 to 400 base pairs).

For quality control purposes, individual non-degraded and degraded DNA samples were amplified with both GlobalFiler (Thermo Fisher Scientific) and PowerPlex Fusion 6C (Promega, Madison, WI, USA) per manufacturer’s recommendations, targeting 1.0 ng DNA input based on the dPCR quantitation results and were run on a 3500xL genetic analyzer [25,26]. It was determined after STR genotyping that a correction factor for the amount of degraded DNA was necessary to make peak heights more comparable at the higher molecular loci. To determine the correction factor, peak height data from the 1.0 ng non-degraded sample was compared to the degraded sample at all alleles < 100 bp. The average ratio of peak heights in nondegraded/degraded was calculated, resulting in a correction factor of 2.75.

### 2.3. Preparation of DNA Mixtures

Evaluation of length-based allelic overlap was performed in two ways. First, an allele sharing ratio (ASR) was calculated for each potential combination of three-, four-, or five-person mixtures by counting the number of alleles across 23 autosomal loci in each combined set of donors and by dividing the number of alleles in the single-source profiles of the same donors. Second, the number of unique alleles per locus was calculated for each potential three-, four-, or five-person mixture, assuming full profiles for each donor. The distributions of the number of alleles per locus were evaluated for all desired combinations of donors (three, four, or five) among these 11 samples, and combinations were chosen to represent different allelic overlap combinations, minor contributor input amounts, and mixture ratio complexity.

**Figure 1:**
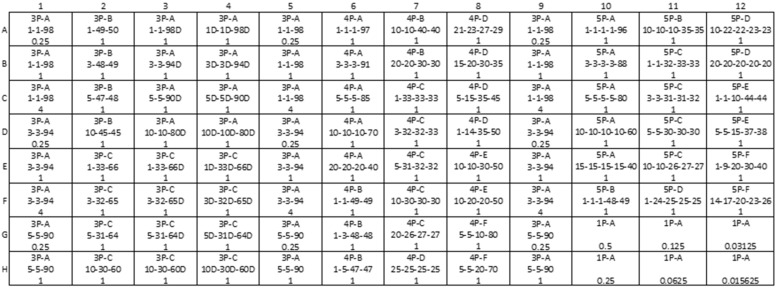
Mixture plate layout. First line in each cell: number of contributors (1P = one, 3P = three, 4P = four, and 5P = five) and sample combination (letters A through F); second line: ratio of each contributor and indication of degradation (D); third line: DNA input in nanograms.

Two working stocks (10 ng/µL and 1 ng/µL) were used to prepare the mixtures (*n* = 74). The mixture tubes were oriented into a plate layout for ease of use in CE STR genotyping and sequencing (Figure 1). The plate layout contains eight samples in triplicate (Columns 1, 5, and 9) of low-level three-person mixtures (two of the three contributors having 1%, 3%, or 5% contribution) with varying levels of input DNA into the sequencing assay (4 ng, 1 ng, and 0.25 ng) to provide information on sensitivity and reproducibility. These replicates were prepared from one stock, which was aliquoted into three sets of tubes. Columns 3 and 4 of the plate layout contain replicate three-person mixture ratios and contributors, with degraded DNA from varying contributors. In the first replicate (Column 3), only the major contributor is degraded and in the second replicate (Column 4), all three contributors are degraded. The remaining three-, four- and five-person mixture ratios appear singly in the plate layout and are composed of non-degraded samples. Finally, the plate layout contains a single-source dilution series ranging from 0.5 ng to 15.6 pg (Rows G and H of columns 10, 11, and 12). The final working concentration for each sample was targeted such that 2 µL of the sample achieved the indicated DNA input amount in Figure 1. The final mixtures were stored at 4 °C until testing. Final mixture dilution calculations for all samples are provided in Supplemental File S1 (https://strbase.nist.gov/Information/Forensic_DNA_Open_Dataset, accessed on 14 February 2025).

### 2.4. STR Genotyping by Capillary Electrophoresis

STR genotyping through CE was performed with PowerPlex Fusion 6C (Promega) and GlobalFiler (Thermo Fisher Scientific) following the manufacturer’s recommended protocol and targeting 1.0 ng of input DNA and separated on a 3500xL using POP-4 polymer (Thermo Fisher Scientific) on a 36 cm capillary array with an injection time of 15 s at 1.2 kV. After data collection, interpretation was performed in GeneMapper IDX v1.6 (Thermo Fisher Scientific) using bins and panels provided by the manufacturers. Allele calls were performed using an analytical threshold of 50 RFU.

### 2.5. Sequencing

Sequencing was performed with three commercial assays targeting varying combinations of auSTR, Y-STR, X-STR, and SNP loci. Libraries were pooled for sequencing according to manufacturer recommendations, resulting in varying numbers of samples in each sequencing run. The plate layout was designed to allow for the sequencing of all 96 samples or three sets of 32 samples, with approximately equal amounts of total DNA input across columns 1–4, columns 5–8, and columns 9–12, and each of these three sequencing runs containing a column of replicates (columns 1, 5, and 9).

The ForenSeq DNA Signature Prep Kit libraries were prepared according to the manufacturer’s recommended procedure [27,28] using DPMB with the DNA input noted in Figure 1 into PCR 1. The flow cell loading procedure was modified by increasing the amount of library pool added to the flow cell from the recommended 7 µL to 10 µL. Libraries were sequenced in sets of 32 following the schema described above for a total of three sequencing runs on the MiSeq FGx Sequencing System (QIAGEN, Hilden, Germany). The resulting sequence data was exported as FASTQ format files.

The Precision ID GlobalFiler NGS Panel v2 kit and Precision ID DL8 Kit were used to prepare libraries according to the manufacturer’s recommended procedure [29] for use with the Ion Chef Instrument (Thermo Fisher Scientific), with the DNA input noted in Figure 1. Library pools from the Ion Chef were quantified with the Ion Library Taqman Quantification Kit (Thermo Fisher Scientific) on a 7500 Real-Time PCR System for Human Identification (Thermo Fisher Scientific) with normalization calculations performed in Excel. Libraries were normalized to 50 pmol/L, and sequencing template reactions were prepared using the Precision ID Chef and Sequencing Kit (Thermo Fisher Scientific) and Ion 530 Chips (Thermo Fisher Scientific). Samples were sequenced in batches of 32 samples per 530 chips according to the schema described above (3 batches of 32 samples processed at a time) and run on an Ion S5XL (Thermo Fisher Scientific). The resulting sequence data was exported from the Ion Torrent Suite Software v5.12 (Thermo Fisher Scientific) as FASTQ format files.

The PowerSeq 46GY Kit (Promega) was used to amplify target STR regions according to the manufacturer’s recommended procedure [30], with the DNA input noted in Figure 1. Library preparation used the TruSeq PCR Free kit (Illumina, San Diego, CA, USA) and IDT for Illumina UD Indexes (Illumina) following Promega instructions. Library construction targeted 500 ng of purified PCR product input. Some amplification reactions did not meet the desired yield, in which case a volume of 25 µL was used to add all available purified PCR products to the initial end-repair reaction in library preparation. After completion, libraries were assayed by quantitative PCR (qPCR) using the Promega PowerSeq Quant MS System (Promega) on a 7500 Real-Time PCR System for Human Identification (Thermo Fisher Scientific). Data from qPCR was used to normalize the libraries to 4 nmol/L, and the final portion of the Promega protocol was followed to begin sequencing the full set of 96 samples in one sequencing run on a MiSeq FGx Sequencing System (QIAGEN) in RUO mode using a MiSeq Reagent Kit V3 cartridge (600 cycles) (Illumina). The resulting sequence data was exported as FASTQ format files.

### 2.6. Stability Testing and Quality Control

Preliminary stability testing using a single-source sample was performed prior to preparing the mixtures to confirm the storage conditions of the perfluoroalkoxy fluoropolymer (PFA) vials. Concentrations down to 0.016 ng/µL were tested with dPCR and STR genotyping, and remained stable.

Multiple methods of quality control were implemented for the final sample set to determine concordance and to assess if the level of coverage and mixture proportions were empirically accurate. dPCR and CE STR typing were used for quality control testing of the final sample set. CE STR genotyping was subjected to a secondary quality check using STRmix v2.8.0 to ensure that the observed mixture proportions approximated the expected mixture proportions prior to sequencing the samples.

FASTQ files from sequencing with these three different methods were processed via an in-house analysis pipeline using STRait Razor v3.0 (SRv3) for the analysis [31]. SRv3 uses a configuration file specific for each kit, which was modified to approximate the ranges given in the Forensic Sequence STRucture Guide hosted at https://strider.online/nomenclature (accessed on 22 October 2024). The results were processed by removing sequenced alleles with less than 10× coverage. A sequence-based concordance check for the mixture samples was conducted on the resulting output from the analysis pipeline on the three sequencing kits at the 22 auSTR loci in common. The sequence-based alleles for the single-source samples were merged in silico to generate the “expected” mixture profiles (containing all potential alleles regardless of mixture proportions), and the number of potential sequence-based alleles were counted. The “expected” mixture sample sequences were compared to the observed sequences in each mixture to determine a “matched” count (the number of potential alleles that were observed). A “match/expected” ratio was then calculated. Additionally, counts of unique sequences and overall number of sequences were determined for each locus.

A subset of sequence data was also deconvoluted using STRmix NGS v1.0.0.36 Research and Validation software (Institute of Environmental Science and Research, Aukland, New Zealand) with default MCMC settings. This analysis was performed on all non-degraded three-person mixtures sequenced with the ForenSeq DNA Signature Prep Kit with expected input DNA quantities of 1 ng. The ForenSeq Universal Analysis Software (UAS) v1.3.6897 sample detail reports were imported into STRmix, and analysis was performed using default thresholds. D22S1045 was excluded from mixture interpretation due to reported high stuttering and heterozygote imbalance, which was observed during this study [18,32,33].

## 3. Results

### 3.1. Mixture Plate Design-Allelic Overlap

**Figure 2:**
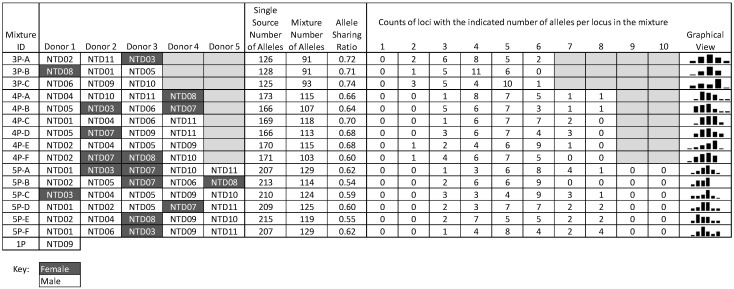
Details of the three-, four-, and five-person sample combinations selected for the mixture plate design. Sample identifiers (e.g., NTD02) for donors included in each mixture (e.g., 3P-A), allele sharing ratio (ASR, the number of expected alleles in the separate single-source samples divided by the number of expected alleles when the samples are combined in the mixture), and counts of loci with the indicated number of alleles expected per locus (numerical and graphical view).

In the analysis of all possible allelic overlap among these 11 samples, the calculated three-person mixture ratios ranged from 0.64 to 0.82 with an average of 0.74; four-person mixture ratios ranged from 0.56 to 0.74 with an average of 0.65; five-person mixture ratios ranged from 0.52 to 0.64 with an average of 0.59 (Supplemental File S2, https://strbase.nist.gov/Information/Forensic_DNA_Open_Dataset, accessed on 14 February 2025). As expected, the mixture ratio is inversely proportional to the number of contributors due to increased allele sharing. Combinations that were representative of typical distributions among the 11 samples were chosen. The allelic overlap of mixture combinations chosen for this mixture set is shown in Figure 2, including the contributor combinations (including biological sex), mixture ratios, and number of alleles per locus.

### 3.2. Sample Degradation

**Figure 3:**
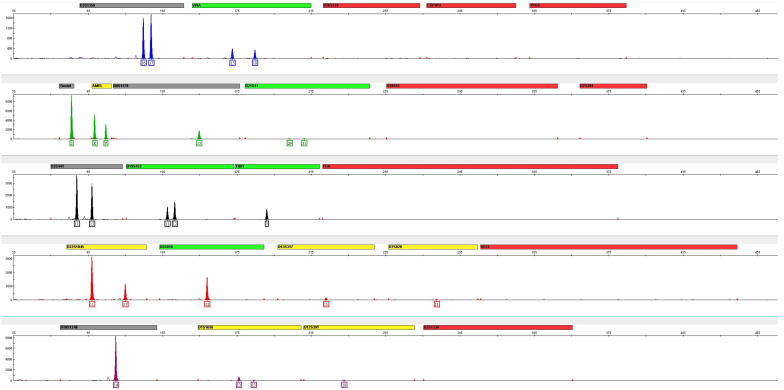
Preliminary degradation data with 1.0 ng of DNA input for GlobalFiler prior to establishing the correction factor. The profile shows significant allelic dropout at the higher molecular weight loci, which is not expected for this level of sonication.

After STR genotyping, the results demonstrated a need to increase the amount of degraded DNA to empirically satisfy an STR profile representing 1.0 ng of DNA input. There is a significant drop at the higher molecular weight loci (Figure 3). Due to the dPCR amplicon having a 67 bp size, the dilution of the samples based on the dPCR quantitation did not accurately represent the concentration of the samples when examining amplicons in the 100 to 400 bp size range. Thus, when targeting 1.0 ng of non-degraded DNA, 2.75 times the amount of degraded DNA input was added to make the peak heights more comparable, especially at the higher molecular weight loci.

### 3.3. Mixture Quality Check

The dPCR quantitation results and their deviation from the expected values targeted were calculated for the final set of mixture samples. All samples deviated by less than 0.1 ng from the expected value except for the degraded samples. Due to the correction factor (e.g., 2.75×) employed for degraded DNA, dPCR results were approximately 1.1 ng higher on average (0.2 ng to 1.4 ng range) for the degraded samples.

Table 1 shows the targeted (expected) mixture proportions, observed mixture proportions via CE (as determined via STRmix v2.8.0 deconvolution), and the difference between expected and observed proportions. The STRmix deconvolution of the STR CE data was primarily performed to determine the accuracy of the mixture ratios developed.

### 3.4. Sequencing Results

Sequencing run metrics and sample-specific information (e.g., samples that exhibited unexpectedly low read counts and samples that were re-sequenced) were documented in the README files associated with the corresponding data files in the Forensic DNA Open Dataset. Quality parameters (e.g., cluster/loading density) were generally in the expected range for each kit and platform. Sample-specific sequencing results for each platform are discussed in detail below.

**Figure 4:**
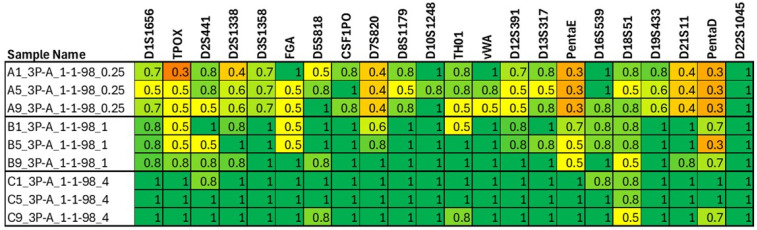
Heatmap indicating the ratio of matched (detected) alleles and expected (potential) alleles for undegraded replicates of 1:1:98 three-person mixtures with 0.25 ng, 1 ng, or 4 ng of input DNA in the PowerSeq 46GY system. Dark green cells denote that all expected alleles were detected, and light green, yellow, and orange cells denote that increasingly fewer expected alleles were detected as the colors move from green to orange. As the level of input DNA increases, the number of detected alleles also increases. Full results can be found in Supplemental File S3.

**Figure 5:**
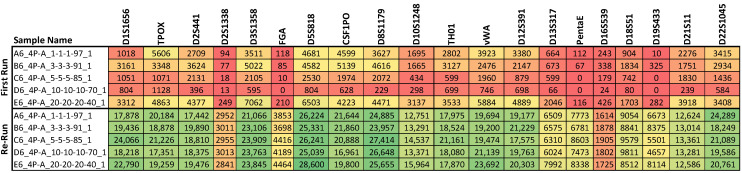
The total coverage per locus for five mixture samples, all with 1 ng input DNA in the Precision ID GlobalFiler NGS Panel v2. Due to lower-than-expected sequencing coverage in the first sequencing run (“First Run”), these samples were re-sequenced (“Re-Run”) in smaller batches, improving coverage. Dark green cells denote higher cover, and light green, yellow, orange, and red cells denote increasingly lower coverage as the colors move from green to red.

The results from the sequence-based allele concordance check for the mixture samples (conducted for all three sequencing kits) can be found in Supplemental File S3 (https://strbase.nist.gov/Information/Forensic_DNA_Open_Dataset, accessed on 14 February 2025). The matched/expected ratio for each sequencing kit gives a sense of how many expected (potential) alleles were matched (detected) in each mixture sample. The results show the anticipated trends of fewer contributor alleles detected in more disparate mixtures and in samples of lower total input DNA amount (Figure 4). Additionally, when samples were re-sequenced due to lower-than-expected coverage, both datasets were included in this check, and the results were as anticipated: lower locus coverage results in a lower matched/expected ratio (Figure 5).

Some kit-specific differences in locus performance may be indicated by these results; however, this analysis was performed as a basic quality check only. Further data exploration is encouraged. Note that this check was attempted only for the 22 autosomal STR loci that are common across all three sequencing kits. For Precision ID GlobalFiler NGS Panel v2, this concordance check excluded the D7S820 and Penta D loci due to a lack of optimization of the in-house bioinformatic pipeline. Future improvements to the in-house pipeline are expected to improve results for this kit.

The ForenSeq UAS sample reports were imported into STRmix NGS v1.0.0.36 Research and Validation as an additional method to examine the expected and observed mixture proportions. The difference between expected and observed proportions was calculated (Table 2), and the results are also in line with expectations.

## 4. Discussion and Conclusions

This publicly available forensic DNA sequence mixture dataset was developed to support the efforts identified by the SWGDAM Next-Generation Sequencing Committee. Its goal is to contribute open datasets to method developers’ needs for bioinformatics and statistical interpretation of complex mixtures. The samples that comprise the dataset were evaluated to allow for varying levels of complexity in DNA quantity, quality, number of contributors within a mixture, and differing mixture proportions.

Digital PCR was used to determine the concentration of the materials. A preliminary quality check of the mixture samples was performed with a CE-based autosomal STR assay and probabilistic genotyping software prior to sequencing. Once the samples were sequenced, additional quality checks were performed on the autosomal STR sequence results with a research version of sequence-based probabilistic genotyping software, as well as in-house tools. The downloadable datasets include all markers within each kit (Y-STR, X-STR, SNP, as applicable); however, additional marker types were not used in quality control checks as tools or methods were not readily available. We encourage additional analysis of these datasets, and we welcome community feedback.

The FASTQ data files for the three sequencing kits for the mixtures and single-source samples and corresponding metadata, along with .hid files from the STR genotyping, are publicly available at doi.org/10.18434/M32157. The data is located as part of the Forensic DNA Open Dataset and is accompanied by README files with additional information about each of the datasets and files from this mixture sample set. These README files contain information on sample re-runs, low coverage, and other anomalies present in the data that may be impactful to the user. The mixture data files may be used to assist the development of algorithms for mixture deconvolution using NGS datasets, to be used as internal training sets by laboratories, or drive new bioinformatic tools for the forensic DNA community. With no existing public datasets for mixture deconvolution available to the forensic DNA community, the goal of this dataset is to offer an exploratory set of data to both software developers and laboratories to assist in their bioinformatic needs. As of January 1, 2025, there have been a total of 761 file downloads for the data related to this study in the NIST Forensic DNA Open Dataset.

## Figures and Tables

**Table 1:** Target mixture proportions, STRmix v2.8.0 assigned mixture proportions for PowerPlex Fusion 6C (PPF6C) STR genotyping data, and the difference between the target and STRmix mixture proportions.

Sample Name	Target Proportions (%)			PPF6C CE STRmix Proportions (%)			Target—STRmix Proportions (%)		
B1_3P-A_1-1-98_1	1	1	98	0.8	1.4	97.8	−0.2	0.4	−0.2
B5_3P-A_1-1-98_1	1	1	98	0.9	1.7	97.4	−0.1	0.7	−0.6
B9_3P-A_1-1-98_1	1	1	98	0.9	1.5	97.7	−0.1	0.5	−0.3
E1_3P-A_3-3-94_1	3	3	94	2.0	4.0	94.0	−1.0	1.0	0.0
E5_3P-A_3-3-94_1	3	3	94	3.0	4.0	93.0	0.0	1.0	−1.0
E9_3P-A_3-3-94_1	3	3	94	3.0	5.0	93.0	0.0	2.0	−1.0
H1_3P-A_5-5-90_1	5	5	90	5.0	6.0	89.0	0.0	1.0	−1.0
H5_3P-A_5-5-90_1	5	5	90	3.0	9.0	87.0	−2.0	4.0	−3.0
H9_3P-A_5-5-90_1	5	5	90	4.0	6.0	90.0	−1.0	1.0	0.0
A2_3P-B_1-49-50_1	1	49	50	2.0	46.0	52.0	1.0	−3.0	2.0
B2_3P-B_3-48-49_1	3	48	49	3.0	44.0	53.0	0.0	−4.0	4.0
C2_3P-B_5-47-48_1	5	47	48	5.0	41.0	53.0	0.0	−6.0	5.0
D2_3P-B_10-45-45_1	10	45	45	12.0	41.0	47.0	2.0	−4.0	2.0
E2_3P-C_1-33-66_1	1	33	66	1.0	34.0	65.0	0.0	1.0	−1.0
F2_3P-C_3-32-65_1	3	32	65	1.0	33.0	66.0	−2.0	1.0	1.0
G2_3P-C_5-31-64_1	5	31	64	4.0	30.0	66.0	−1.0	−1.0	2.0
H2_3P-C_10-30-60_1	10	30	60	10.0	32.0	58.0	0.0	2.0	−2.0

**Table 2:** Target mixture proportions, STRmix NGS v1.0.0.36 Research and Validation assigned mixture proportions for ForenSeq DNA Signature Prep Kit sequencing data, and the difference between the targeted and STRmix mixture proportions.

Sample Name	Target Proportions (%)			ForenSeq STRmix NGS Proportions (%)			Target—STRmix NGS Proportions (%)		
B1_3P-A_1-1-98_1	1	1	98	2.2	3.8	94.0	1.2	2.8	−4.0
B5_3P-A_1-1-98_1	1	1	98	5.5	7.2	87.3	4.5	6.2	−10.7
B9_3P-A_1-1-98_1	1	1	98	2.3	3.2	94.6	1.3	2.2	−3.4
E1_3P-A_3-3-94_1	3	3	94	3.0	4.4	92.6	0.0	1.4	−1.4
E5_3P-A_3-3-94_1	3	3	94	2.0	4.7	93.3	−1.0	1.7	−0.7
E9_3P-A_3-3-94_1	3	3	94	3.1	5.6	91.3	0.1	2.6	−2.8
H1_3P-A_5-5-90_1	5	5	90	4.6	6.4	89.0	−0.4	1.4	−1.0
H5_3P-A_5-5-90_1	5	5	90	5.9	8.0	86.1	0.9	3.0	−3.9
H9_3P-A_5-5-90_1	5	5	90	4.0	6.2	89.8	−1.0	1.2	−0.2
A2_3P-B_1-49-50_1	1	49	50	2.8	46.0	51.3	1.8	−3.0	1.3
B2_3P-B_3-48-49_1	3	48	49	3.3	43.2	53.5	0.3	−4.8	4.5
C2_3P-B_5-47-48_1	5	47	48	4.3	45.1	50.6	−0.7	−1.9	2.6
D2_3P-B_10-45-45_1	10	45	45	8.0	42.0	49.9	−2.0	−3.0	4.9
E2_3P-C_1-33-66_1	1	33	66	3.2	33.5	63.3	2.2	0.5	−2.7
F2_3P-C_3-32-65_1	3	32	65	4.0	29.9	66.1	1.0	−2.1	1.1
G2_3P-C_5-31-64_1	5	31	64	5.0	30.3	64.7	0.0	−0.7	0.7
H2_3P-C_10-30-60_1	10	30	60	8.9	37.5	53.6	−1.2	7.5	−6.4

## Data Availability

Supporting information can be downloaded at: https://strbase.nist.gov/Information/Forensic_DNA_Open_Dataset (accessed on 14 February 2025) and data associated with the manuscript may be found at: doi.org/10.18434/M32157.

## Supplementary Materials

The following supporting information can be downloaded at: https://strbase.nist.gov/Information/Forensic_DNA_Open_Dataset (accessed on 14 February 2025).
